# IoT Registration and Authentication in Smart City Applications with Blockchain

**DOI:** 10.3390/s21041323

**Published:** 2021-02-13

**Authors:** Célio Márcio Soares Ferreira, Charles Tim Batista Garrocho, Ricardo Augusto Rabelo Oliveira, Jorge Sá Silva, Carlos Frederico Marcelo da Cunha Cavalcanti

**Affiliations:** 1 Computing Department (DECOM), Federal University of Ouro Preto (UFOP), Ouro Preto 35400-000, Brazil; celio@linuxplace.com.br (C.M.S.F.); charles.garrocho@ifmg.edu.br (C.T.B.G.); cfmcc@ufop.edu.br (C.F.M.d.C.C.); 2 Department of Electrical and Computer Engineering, University of Coimbra, 3030-290 Coimbra, Portugal; sasilva@deec.uc.pt

**Keywords:** Blockchain, Smart City, Smart contract, IoT

## Abstract

The advent of 5G will bring a massive adoption of IoT devices across our society. IoT Applications (IoT Apps) will be the primary data collection base. This scenario leads to unprecedented scalability and security challenges, with one of the first areas for these applications being Smart Cities (SC). IoT devices in new network paradigms, such as Edge Computing and Fog Computing, will collect data from urban environments, providing real-time management information. One of these challenges is ensuring that the data sent from Edge Computing are reliable. Blockchain has been a technology that has gained the spotlight in recent years, due to its robust security in fintech and cryptocurrencies. Its strong encryption and distributed and decentralized network make it potential for this challenge. Using Blockchain with IoT makes it possible for SC applications to have security information distributed, which makes it possible to shield against Distributed Denial of Service (DDOS). IoT devices in an SC can have a long life, which increases the chance of having security holes caused by outdated firmware. Adding a layer of identification and verification of attributes and signature of messages coming from IoT devices by Smart Contracts can bring confidence in the content. SC Apps that extract data from legacy and outdated appliances, installed in inaccessible, unknown, and often untrusted urban environments can benefit from this work. Our work’s main contribution is the development of API Gateways to be used in IoT devices and network gateway to sign, identify, and authorize messages. For this, keys and essential characteristics of the devices previously registered in Blockchain are used. We will discuss the importance of this implementation while considering the SC and present a testbed that is composed of Blockchain Ethereum and real IoT devices. We analyze the transfer time, memory, and CPU impacts during the sending and processing of these messages. The messages are signed, identified, and validated by our API Gateways and only then collected for an IoT data management application.

## 1. Introduction

We currently live in an urgent need to develop new and disruptive security solutions capable of supporting the long-awaited mass adoption of IoT devices promoted by the arrival of 5G. This IoT device is already widely available for purchase, using API, protocols, and server infrastructure is often proprietary. An absence of standard and consensus among manufacturers leads to the exchange of data in an unstandardized network architecture.

IoT devices being set in urban areas as pipes, energy sources, sewage, waste bins, temperature, and the high mountain makes physical access to devices challenging. A simple battery change or firmware update in some places requires difficult operational effort, sometimes being impossible or costly to complete quickly. Based on this awaited scenario, it is impossible to use the security features that are provided by default; new layers and security proposals to exchange messages from outdated IoT devices and Apps are necessary. When extracting data from an urban area, the most common scenario is to use IoT devices to receive data from the most diverse sources and devices [1].

The Smart Cities (SC) is a fertile field for these applications. An urbanized space is used to obtain information, as well as prevent and manage urban problems, with IoT being an efficient way of data extraction [2]. An SC App had a significant percentage of data veung characterized as sensible; this makes security a prerequisite. This new IoT hardware is often unstandardized and unreliable. Its low cost and simplicity of configuration make scale management a challenge. The SC App is a strong candidate to be a pioneering use of mass IoT 5G connected, thus improving an unsafety design’s costs and risks.

The security characteristics of the Blockchain allow for some of these challenges minimized. Blockchain allows robust security to receive data from participants that can often be unreliable. The possibility of writing a routine using Blockchain entities by Smart Contracts allows for the development of new applications, such as the IoT devices, spread in an urban environment, like cities.

Blockchain has the properties and robust security characteristics to help in this challenge. Although it has achieved hype in recent years, it has only been put to the test in applications that are restricted to financial markets, with cryptocurrencies being its biggest highlight. However, when evaluating IoT use cases, it is necessary to consider some of the limits and possibilities, proposing new connectivity layouts, frameworks, and consensus protocols.

The Blockchain has distributed infrastructure, and this makes it scalable and resilient to Distributed Denial of Service (DDOS) attack [3]. SC Apps’ usual security is centralized security architecture, which is inefficient for unpredictable growth applications, focusing on attacks that are concentrated on a weak point, have low scalability, and are ineffective in receiving an increasing number of simultaneous transactions.

The IoT security problems justify our research, and the possibilities for developing Blockchain-based applications SC are very promising. Countries, like the United Arab Emirates, the US, and the UK, already use these in government and the public sector. For example, Dubai plans that all public services be Blockchain-based by 2020 [4].

A typical SC IoT App problem is registry devices for verifying the source of sensible urban data.

The Blockchain characteristics make it possible to receive signed and identified payloads from unknown and untrustable IoT devices, often with outdated firmware. We hypothesize that we can use Edge/Fog computing strategies and Blockchain to provide a reliable, robust, and decentralized security environment. It makes it possible to verify the origin and content of data from an IoT device.

We propose a model for identifying and registering an IoT device inserted in an SC App in this work. We develop an API Gateway to verify the identity and authenticate sign messages received by IoT devices, using Blockchain and Smart Contracts.

This strategy of security use network paradigms Edge and Fog Computing. These paradigms have potential in IoT implementations, because they are efficient and economical, even with low data transfer rates. They are suitable for demands where the application’s intelligence is close to the information producing devices. The main idea is to send consolidated, signed, verified, and identified messages to the endpoint API that is located in cloud or management network layers.

This is discussed, as a SC IoT App can benefit from Blockchain facing the conventional technologies. We have seen that by addressing a decentralized structure, some applications already reduce operating costs, reduce risk, and increase trust.

To test our hypothesis, we develop API gateways **IoT Edge API Gateway** and **Blockchain API Gateway**. These API gateways run in Edge and Fog Computing, and they sign and verify the IoT message’s authenticity, using Smart Contracts deployed in an Ethereum Blockchain. The **Blockchain API Gateway** call the Smart Contracts of the project IoT Device Management [5] a Decentralized Applications (Dapp). We present a testbed using real devices running the **IoT Edge API Gateway** to validate messages before sending to a server running the project IoT Framework Engine [6]. This objective of the testbed is to represent a typical SC IoT App scenario.

### Contribution and Organization

How to contribute to this work, we can list:

- The **IoT Edge API Gateway** project is a daemon running on an IoT device, which is responsible for assuming two sensors receiving messages and preparing a payload containing assurance and metadata that are used to aid in the verification of authenticity;
- the **Blockchain API Gateway** project, is a daemon running in bastion that protects the application network. The daemon receives the **IoT Edge API Gateway** payloads. If the sender’s authenticity is verified, the message follows the protected application network’s data management server address;
- a discussion about Blockchain and Smart Contract in SC Apps; and,
- a testbed with real devices and an IoT data management project as proof of concept.

In Section 2, we show the technologies and concepts that are necessary to understand we work, and Section 3 presents the related works. Section 4 presents the advantages of a decentralized security management system. In Section 5, we discuss the challenge and scenarios of a Blockchain to IoT SC App. In Section 6, our contribution’s architecture is detailed, and Section 7 shows the testbed used for the experiment and its results. Finally, the conclusion in Section 8.

## 2. Background

In this session, we present the leading technologies that are used in our research. We use Blockchain as a repository for Smart Contracts and data that are responsible for identifying and validating IoT devices. The open-source Ethereum is the Blockchain network we use, which is the infrastructure for deploying Smart Contracts. The functions that are found in the Smart Contracts used by this work are the registry rules and validation of a device. The Merkle Tree data model is the latest technology covered. When registering the IoT device, a set of metadata is registered that helps identify it later. A calculated Merkle root hash using this set of metadata is stored in the Blockchain for future reference in the validity of metadata informed by IoT devices.

### 2.1. Blockchain

Blockchain is a ledger in which all of the transactions are signed and replicated among the network nodes that do not need a trusted central intermediary authority.

It is a recent technology and has not yet been tested in all scenarios. It is a robust adoption solution in financial services and cryptocurrency. As a distributed technology, it presents the scalability and immutability required by many modern applications.

Transaction auditing dais made possible, because it stores a consistent transaction history. This feature is essential for IoT applications that can receive data from untrusted remote devices. This feature allows for reliable store data, as it cannot be changed or deleted. It has a distributed, decentralized network paradigm with nodes in P2P (peer to peer) with a highly scalable nature. The strong security is based on the high computational cost consensus algorithm and elliptic curve cryptography, allowing nodes to store the transaction in blocks without trust between them.

These transactions in Blockchain can be verified anywhere and at any time, which allows a secure, immutable, and auditable transaction history, thus decreasing the possibility of transaction fraud.

Merkle trees are used for checking the content and consistency of data, where the transactions are a binary hash tree where each leaf node is the hash of a transaction, and each non-leaf node’s value is the hash of the nodes below it.

### 2.2. Smart Contracts

We have Smart Contracts, a concept introduced by Nick Szabo in 1994, to run programs on the Blockchain [7]. These are programs deployed in a Block as a regular transaction. To call necessary know the ABI interface a the Blockchain address, lead possible to build complete programs stored and using Blockchain’s security resources [8]. These programs are Dapp, full programs without dependencies of centralized infrastructure and resilient and robust security.

A typical scenario in solutions IoT to an SC is network architect Edge and Fog Computing. These network paradigms mean that most IoT data can be consolidated, processed in the device, before being transmitted to the cloud network.

The difference between Fog Computing and Edge Computing is the network position in which processing occurs. At Fog Computing, the intelligence is at the Gateway node with the internet backbone, and, at Edge Computing, it is the device that processes the data before sending it.

### 2.3. Ethereum

For the development of Smart Contracts, we have the Ethereum Blockchain. The Open Source project is currently listed as one of the tools that have the most significant disruptive impact and is the basis for the operation of other projects of this characteristic. In cryptocurrency popularity, Ethereum is only behind Bitcoin [9]. Smart Contracts on Ethereum is a Turing Complete language and it can be written and compiled in languages like Solidity. It is store as bytecode in a block, and its execution occurs in an Ethereum Virtual Machine (EVM).

Ethereum EVM is a sandbox, and it provides an execution environment in that nodes run the same code. The execution code in Ethereum is called Gas, and each Ethereum transaction must specify a maximum Gas limit. It is void, for instance, that Smart Contract does run forever [10].

The strong security of Ethereum Blockchain and the ability to write routines make it potential for applications that are distributed in a scattered and unknowing space as the IoT Apps in SC scenaries. Its distributed consensus algorithms and quick adherence of the open software community to the project make Ethereum an excellent candidate for our proof of concept. Receiving data from edge devices without confidence should be a significant challenge for the win when extracting data and sending data to the cloud environment simultaneously in legacy and unsecured API.

### 2.4. Merkle Tree

**Figure 1:**
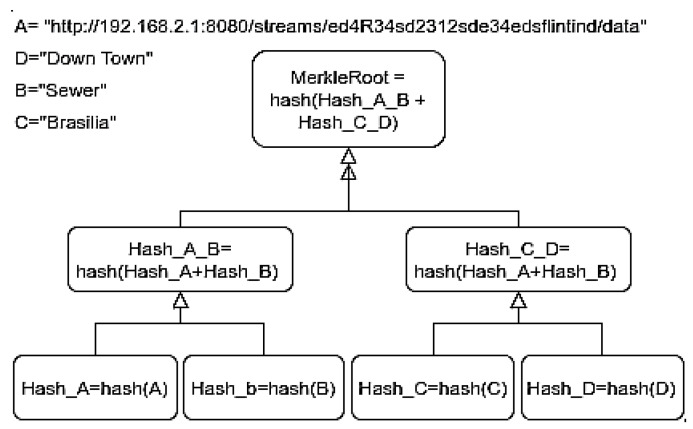
Testbed Mekle Tree metadata.

The Blockchain uses the Merkle tree to verify transactions’ consistency, as in Figure 1. This data structure is just a complete binary tree of hashes [11]. Each leaf node is a hash of an object, and each non-leaf node is a hash of its previous hashes.

The proofs of a Merkle tree are used to decide if a piece of data belongs to the tree, and it is consistent with a set of data without having to reveal it.

For example, in order to verify data [C] in Merkle Root, we use the function, hash [C], which results in Hash_C. Furthermore, to validate whether C belongs to the Merkle tree, it is unnecessary to reveal it. Hash_A_B is the Hash_A when hashed with Hash_B, Hash_C_D is the Hash_C when hashed with the hash of unknown D, Hash_D. Hash_C_D hashed with Hash_A_B result in the Merkle Root Hash_A_B_C_D.

We use Hash_D, Hash_C_D, and Hash_A_B without revealing C or any data in order to prove that the C data is present in the Merkle tree.

With these, we can obtain Hash_A_B_C_D, therefore proving that Hash_C was part of the Merkle tree, which implied that data C was, in fact, part of the universal data set [A, B, C, D].

The hash function generally uses SHA-2, although others can also be used. Ethereum uses Keccak-256 belonging to the SHA-3 family of hash functions.

## 3. Related Work

In this session, we discuss the works related to our research. Table 1 summarizes the techniques, problems, and contributions of the studies.

Blockchain can be applied in various application domains and sectors of our society, covering almost all aspects of business, industry, finance, and governance, among others. Surveys, such as [12], concentrate the works that address Blockchain applied to information systems and their security aspects. The work [13] studied the challenges and opportunities of using Blockchain as a database for IoT Apps.

We can find a pros and cons analysis of Blockchain integration’s possibilities with IoT in [14]. The work combines Blockchains and IoT, and emphasizes the power of this union of technologies. It can be quite powerful, where Blockchain provides resilient and truly distributed peer-to-peer systems, the ability to interact reliably and without auditing. By approaching Smart Contracts, the work shows that it is possible to automate complex processes, with IoT devices being the contact points with the physical world. In the article, the combination of Smart Contract, Blockchain, and IoT is a breakthrough in automating workflows in new and unique ways, which enables cost-effective and time-saving cryptographic verifiability. Its conclusion estimates that the integration of Blockchains into IoT applications causes significant transformations in various sectors of the economy, bringing new business models and rethinking systems and process implementation.

As in our proposition, article [15] discusses the implementation of a Smart City that is integrated with the Blockchain providing IoT devices with a secure communication platform. The article proposes a framework that securely integrates the physical layers, IoT communication, and application interface. Differently, our work focuses on investigating the impacts of using Blockchain in sending the message to an SC API after registration, identification, and recognition of IoT devices.

The work [16] addresses the security requirements for an IoT Blockchain network, and discusses how these can be satisfied through Smart Contracts. This article presents a prototype in which all operations that are related to Blockchain use an API gateway. The paper addresses the main challenges associated with IoT devices’ security and trust in Blockchains, presenting a design of a Global IoT information distribution system using Blockchain.

Some studies use the Fog Computing approach in their IoT application architectures, as well as our research. They show the challenges of processing as much information as possible at the edge of the network [17,18]. We use this paradigm. It allows for sending consolidated data to the cloud, isolating the network’s segments with bandwidth economy, an essential feature to efficient IoT applications.

Edge computing based on cooperation and collaboration is proposed in [19] to share resources and deliver services. An incentive-based mechanism is adopted to offer a reward for the participant in the process using Blockchain.

The research [24], discusses data privacy and the benefits of applying Edge Computing and Blockchain in Industrial IoT (IIoT) scenarios, implementing experiments in Ethereum to evaluate security, performance, and energy efficiency.

The study [20] presets this discussion. The authors use the open-source projects, TensorFlow, Docker, and Kubernetes, to implement an edge data analysis platform in the Fog Computing paradigm and an IoT-fog integration utilizing Message Queuing Telemetry Transport (MQTT).

The work [21] approach the security and revocation of data and access for smart Factories services in Industrial IoT. The Blockchain is used in security management to block and revoke access to malicious users, responsible for the identity authentication, public keys, user attribute sets, and revocation lists. The work proposed an attribute-based access control scheme and protocol for the smart factory supporting traceability and revocation over a Bilinear Diffie–Hellman assumption. This result of work is schemes that optimize the size and overhead during the public key generation, data encryption, and data decryption stages.

The paper [22] investigates its energy consumption and performance. In the discovery phase, the MQTT server is a Fog node responsible for enabling/disabling the Bluetooth Low Energy (BLE) interfaces of devices, monitoring their trajectories, energy efficiency, and scalability. The use of AI PnP models in the Edge Computing paradigm and an analysis of the impacts of their usage on SC ecosystems are explored in [23].

The work [5] develops a system based on Ethereum to identify and authenticate IoT devices through Smart contracts. The testbed [25] published on GitHub uses a web interface for this registration of devices, and we use its platform for **Blockchain API Gateway** and **IoT Edge API Gateway** development.

## 4. Decentralized Management Security

Centralized security systems, in which we find user records, passwords, user access keys, and other artifacts, have a latent weakness. Even with auditing and governance rules, these centralized systems are not guaranteed to change data without the user’s exclusive authorization.

Centralization exposes a single point of failure; the chance of an attack as DDOS succeeding increases, which makes these systems vulnerable to this familiar and routine cyber attacks [26]. Centralized systems have unavoidable and unstable behavior when receiving them. This makes these SC applications undesirable and intolerable.

Users’ accesses and data are centralized and controlled by managers, the traditional security and infamous systems, despite having audit logs, are not free from undesirable and unsolicited modifications or, many times, unauthorized by users. A centralized security management system is often made by the managers and modified by them at their leisure. There are risks to privacy, as these centralized entities have no strict or guaranteed control over the use of private data, which is often confidential. Health information, shopping preferences, and behaviors that are stored on central servers are not guaranteed to have power over how user data are used or even by whom [27].

There is no explicit guarantee of privacy in a traditional centralized infrastructure. It is unclear who has access to or who is responsible for the data, and it is not possible to have reliable means to track the use of changes in the data. It remains for users of centralized services to trust management entities and their storage capacity and suitability. In some cases, third parties are responsible for processing and storing data and may be deleted or tampered with without explicit authorization [28].

Our proposal for a decentralized authentication and identification of payloads coming from the Edge points of large-scale networks, such as SC Networks, would allow an added security during the receipt of payloads, while using the decentralized and cryptography resources of Blockchain.

Decentralized security management of IoT sensors with Blockchain ensures greater credibility, more substantial transparency, and resilience to SC Apps’ security, given the guaranty of a trust data source [29]. Thus, the Blockchain needs to be fed with some characteristics that identify the edge IoT device as metadata. A strategy for verifying this previously registered metadata is to undertake a Merkle tree with then and store it in a Blockchain. The proof of metadata presence in a Merkle Tree is used for future verification of data validation.

We propose HTTP API Gateways in IoT Device, Edge Computing, Network Gateway, Fog Computing. A local HTTP API running in an IoT device receives the messages from an IoT device. The messages that are received in this local HTTP API are signed and they send payloads to an HTTP endpoint that works as an API gateway. For verification and validation, the payload sent contains the sign message, the IoT firmware, the address of SC web service API, and other metadata of interest that identify the data source’s origin, reliability, and truthfulness of data. On the delivery of payload, the API Gateway checks the message and its signature, metadata, Merkle Tree proof, and firmware on the Blockchain by Smart Contract; if the payload is validated, it is sent to the SC web service API that is extracted from the payload metadata.

## 5. SC APP Scenarios

A centralized SC network security architecture to IoT has several security limitations. An exposition to Denial of Service (DDOS) attacks is a typical example, where a single point of attack on centralized servers results in operation system failure. Regarding privacy, data stored on centralized servers have private information from users regarding health, purchasing preferences, and behaviors, as there is no guarantee of control over how user data is used or even by whom. Data stored in centralized infrastructure are often not explicitly responsible or traceable [30].

Third parties are often responsible for processing the data, deleting, modifying, or tampering without explicit authorization from the user. The volume of data watered by SC Apps doubts whether centralized security servers will be efficient enough to handle the volume of end-to-end transactions, produced by example by IoT devices [31].

Without a distributed and decentralized platform that guarantees security and transparency and mainly traceability of transactions, such as Blockchain, an SC App that uses data that were collected to generate revenue to cities through fees, can be victims of fraud or manipulation. The exchange of goods and services needs to trust and make transactions involving costs or the use of third-party financial resources, requiring trust. These resources are mandatory for low risk and transparency to transactions [32,33].

A decentralized infrastructure does not depend on relying on other nodes, nor does it need a central authority or trusted intermediary to exchange messages. Blockchain is a potential tool for secure and scalable communications in Smart cities applications, and it can make them quickly become a reality [8,15]. Resilient, the Blockchain has an immutable and durable record; the transaction is only complete after a node consensus, with an immense computational effacement to change or delete it being necessary. Additionally, a P2P network is highly scalable. It was created to use cases of cryptocurrencies and has already been widely used in today’s Fintech structures [34]. Together Blockchain and Smart Contract have the requirements to create techniques that minimize the risks inserted when producing data in an SC App.

In use cases where citizens’ IoT devices can collect data, a Blockchain solution becomes essential [35]. It is an ideal solution for human rights issues, such as personal and data privacy, transparency of the Public Power, citizenship, and security. SC IoT Apps can raise important data for sustainable urban development standards when considering the current development and digital transformation occurring during the COVID19 pandemic.

**Figure 2:**
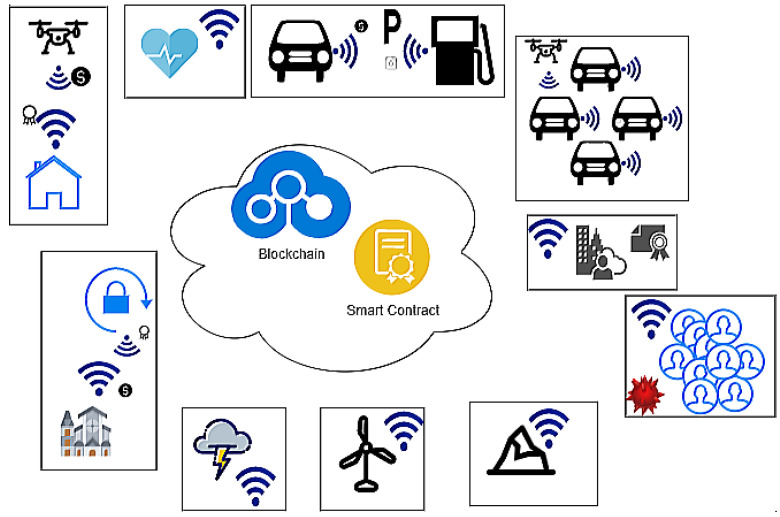
SC, Blockchain and IoT use cases.

The IoT and 5G technology can accelerate the popularization of high transfer rate GPS car sensors, allowing for city traffic data to predict future traffic and congestions [36]. Environmental data, including air quality, temperature, and rainfall, can help citizens or tourists avoid visiting a city point [37]. SC IoT devices, especially those that are placed in hard access locations, is its long-term use. Our API Gatewaycan support SC applications that provide continuous and dynamic views of urban activities. We work to provide a more reliable architecture to receive IoT data while using Blockchain and Smart Contract for verification, even in scenarios where the IoT API is in obsolescence or that have firmware vulnerabilities [38]. Figure 2 presents the main IoT SC cases to use Blockchain and Smart Contracts.

The great majority of this actual SC App uses legacy HTTP API and centralized security management, and we can help to improve its security with this research.

The set of API gateways that we propose in this work can help in the problems that are described in SC Applications presented in Table 2.

Problems that are related to hardware and sensitive data are strong candidates to need extra layers of security. The proposed Blockchain scheme in this paper may be one of the alternatives to be adopted. Its set of security features and decentralization features make it a key technology for the solution.

Outdated firmware on a device can cause an IoT device to be subject to several operational and security problems. Security weaknesses in these devices can cause the leakage of sensitive data or even operational unavailability. Falsifying data coming from these devices can cause credibility and financial damage to city administrations. Our proposition using API gateways with Blockchain for SC Apps is relevant for avoiding message spoofing.

We use the characteristics of IoT devices to validate the payload. Extracting the firmware hash and root hash from the Merkle tree created while using metadata for identification. The messages that are transmitted in the device payload are signed and validated in Ethereum by Smart Contract.

This strategy means that, even if a device has outdated firmware, the previously registered characteristics are checked. This routine is independent of the APIs and security features that come from the device. The falsification of messages is made more difficult by the need for these validations before receiving the message.

## 6. Materials and Methods

In this section, we describe the set of software and projects used to prove the concept of our work. We approach the details of the main functionalities of each project, technologies, and routines that are used to validate and identify the origin of messages sent from the devices.

### 6.1. API Gateways

A gateway that communicates and isolates the production API is one of the architectures currently considered best practice in designing a secure IoT network. These products can be found as API Gateways and they are responsible for isolated environments and organized in separate internal and private business logic functions currently know as Microservice.

A popular solution component used to manage Microservices is API gateways management software, which completes tasks that allow for developers to monitor, transform, and create security layers when exposed to a unique endpoint their internal production Microservices APIs.

We propose API gateways that identity, authentication, and the reputation of messages coming from the IoT devices of an edge network. These gateways would provide an additional security layer using Blockchain and Smart contract. This set of technologies have implicit features for developing security in untrustable environments, such as unknown IoT devices that are spread in a Smart city APP network.

Our API gateways are similar to the already popular authentication tokens, which follow each Payload sent to an API endpoint. Our proposition differs from traditional tokens. We added Blockchain and Smart contract in the background to verify the authenticity of the API Gateway’s payloads before SC App API receives them. API management is a recommended resource, mainly when various devices produce and consume data from different sources, due to SC’s characteristics and the diversity of software and API involved.

In the next Section, we will present our API Gateways and the main interdependent components.

### 6.2. Components of Proposition

Our proposal of API Gateway to validate and identify the payloads that are received from IoT devices is composed of a web frontend for device registration, called **IoT Device Management**, an HTTP API on the IoT device, called **IoT API Edge**, and an HTTP API located at the border of the Fog or Cloud Network of an SC App, called **IoT Blockchain API Gateway**.

#### 6.2.1. IoT Device Management

IoT Device Management is the component that is responsible for the registration of devices in Blockchain using Smart contracts. This component is presented in work [5], and the code is available online in [25].

The IoT Device Management was developed in NodeJs, React frontend ad, the Smart contracts are written in Solidity. Contracts are responsible for registering IoT devices and associating them with their respective owners called Entity. An Entity is an Ethereum account that is represented by its public address.

Here is the list of contracts that make up the basis of IoT Device Management.

- Entitybase, which provides base functionalities for entities and is responsible for all entities and their public attributes.
- DeviceBase provides base functionalities for devices, responsible for associating with an Entity and creating the devices and their properties. Owner, identifier, metadataHash, and firmwareHash.
- DeviceHelper, provides extra functionalities for devices. The function isValidMetadataMember checks whether a provided item is a member of metadata contained in the Merkle tree. The function isValidFirmwareHash, which checks whether a provided firmware hash is equal to the firmware hash device property. isValidEthMessage validates a previously signed message by **IoT Edge API Gateway** using an Ethereum private key.
- signatureBase is the base of functionalities for device signatures, creates and revokes a signature for a device.

**Figure 3:**
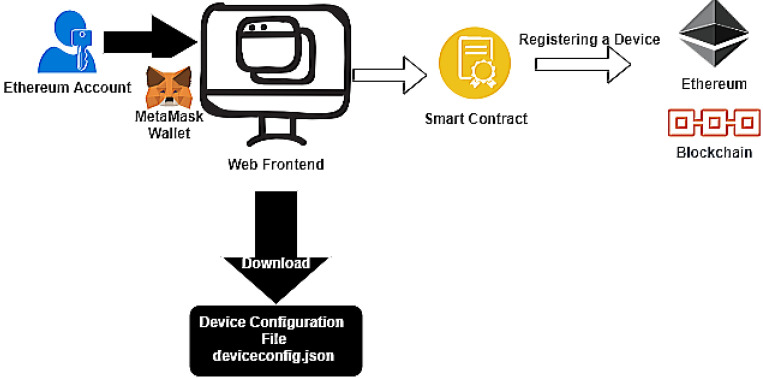
User interacfion using IoT Device Manager.

Figure 3 represents a user’s interaction with the IoT Device Manager to register a device in Ethereum.

In the diagram, we can observe the Metamask [39] Crypto Wallet. It is an Ethereum account manager and it is responsible for securing interaction with applications using web3.js [40] to call Smart contracts.

During the device registration, the user must enter the following fields, the identifier, a set of metadata, and its firmware. The Firmware Hash and root hash of a Merkle tree containing the device’s metadata are calculated and included in the Ethereum registration transaction, as in Figure 1.

**Figure 4:**
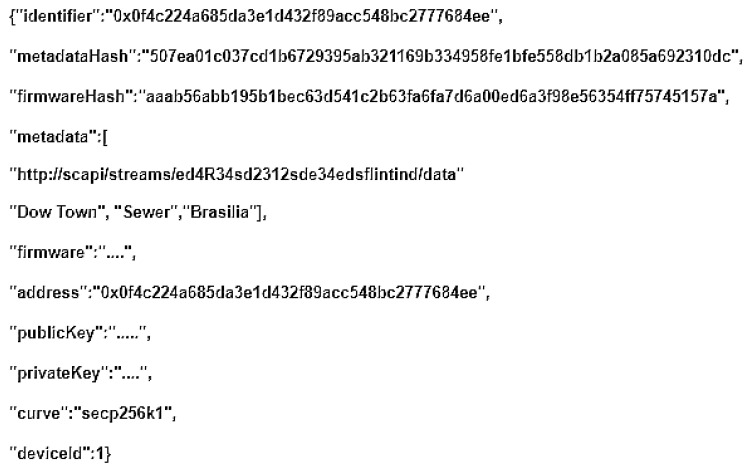
Device Configuration File.

Finally, the Smart contract that registers the new device is called. After its transaction is submitted and mined on the Ethereum network, the new registered IoT device’s configuration file is downloaded, Figure 4.

**Figure 5:**
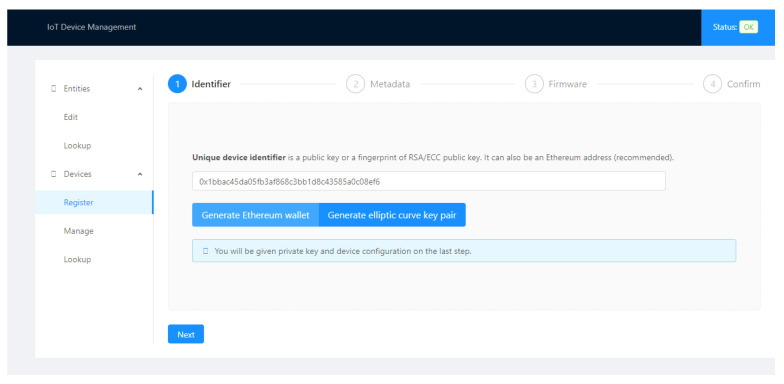
Identifier.

**Figure 6:**
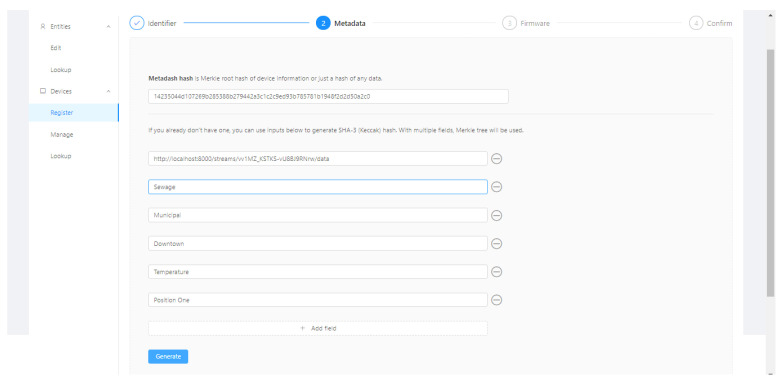
Metadata.

**Figure 7:**
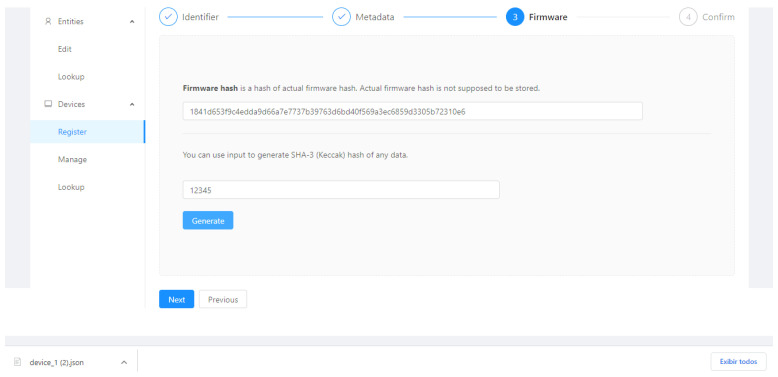
Firmware.

**Figure 8:**
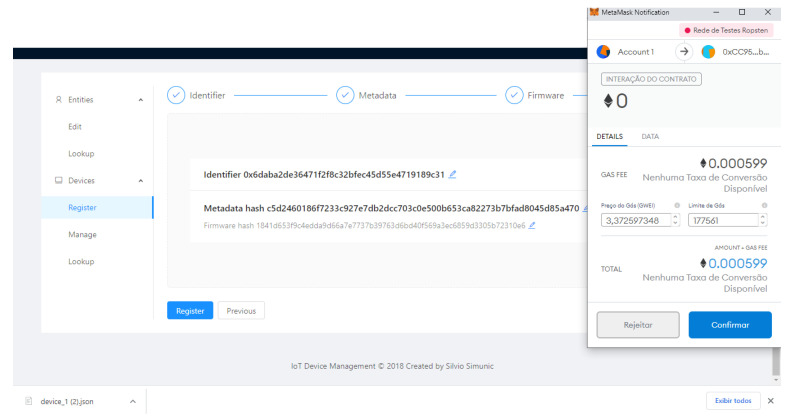
Blockchain Transaction.

Figure 5, Figure 6, Figure 7 and Figure 8 present the sequence of a device registration in IoT Device Management Frontend.

#### 6.2.2. IoT Edge API Gateway

**Figure 9:**
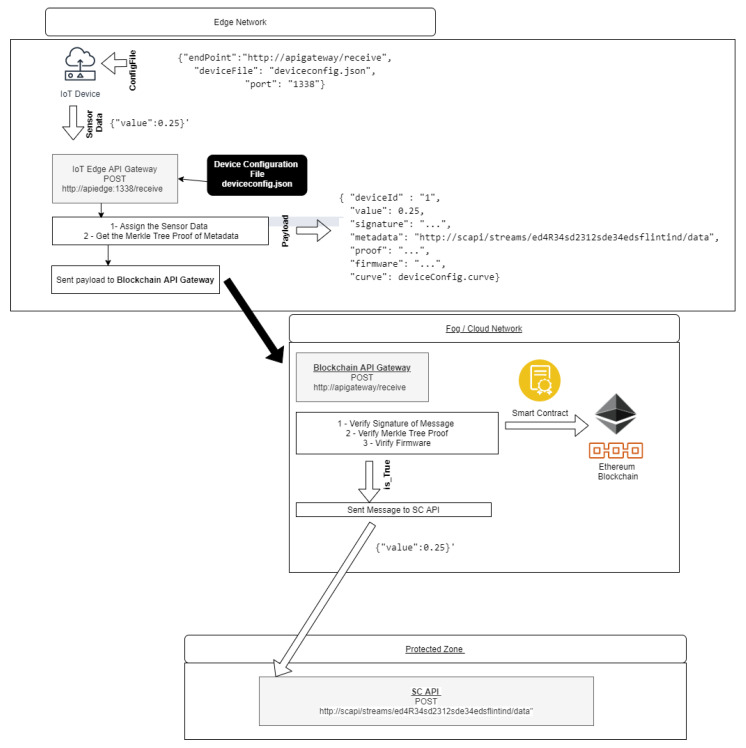
Api Gateway Diagram.

The **IoT Edge API Gateway**, as in Figure 9, is a daemon running on the IoT device, is responsible for signing the message and composing the Payload with other attributes that prove the origin of the message and destines it to the **Blockchain API Gateway**. Developed in NodeJs language, an HTTP Deamon runs locally on the IoT device to receive messages that are extracted from local sensors data. The keys and attributes of IoT Device Management device configuration are used to generate a payload. This Payload contains the device identification, the message, the signed message, the metadata of SC App API HTTP address, the Merkle proof, and the device’s Firmware.

#### 6.2.3. Blockchain API Gateway

The **Blockchain API Gateway**, as in Figure 9, is the component that is responsible for validating and identifying the payloads that arrive from IoT Edge Network devices.

This typical component’s localization is the border of the SC App network. It is a NodeJs HTTP daemon that listens for payloads that arrive from the IoT Edge network. If the message of the Payload is validating, then it sends the message to the SC App API. This API’s internal network address is the first metadata information created when it is registered in Ethereum.

The same Smart Contracts that are used by IoT Device Management are used to validate the IoT API Edge payloads. The API Gateway uses the message, its signature, the metadata (HTTP address of API SC App), its Merkle proof, and the Firmware received from Payload to validate. If validated, the message is forwarded to SC App, and the address used as metadata.

The **Blockchain API Gateway** and **IoT Edge API Gateway** are developed at node.js. The main libraries used are:

- *web3.js*, base library for developing applications that make calls to Ethereum; and,
- *ethereumjs-util*, a collection of utility functions for use with Ethereum

## 7. Experimental Testbed and Results

In order to see the feasibility and dynamics of the solution, we have implemented, as an experiment, a testbed that simulates a Fog/Edge Computing network architecture using auxiliary components and a public Blockchain network.

### 7.1. Experimental Testbed

In our experiment, we use a Docker Desktop on an Intel Pentium Silver N5000 1.10 GHz with 8 GB of RAM for network and Docker containers. Additionally, for experiments with a real IoT device, a Raspberry Pi 3 B +, with Broadcom BCM2837B0 Processor, Cortex-A53 ARMv8 64-bit SoC 1.4 GHz, 1 GB LPDDR2 SDRAM. Table 3 lists the attributes and parameters used in Testbed.

We use Docker and its resources for container management and virtual network to simulate an IoT network environment in contact with its SC API, an Edge/Fog Computing architecture. One of the tools available for the orchestration of containers and networks is Docker Compose. The *DockerCompose* file of the testbed is responsible for deploying three network subnets. The subnets FogNet, EdgeNet, and AppNet have logical and network isolation.

The only container that has contact with IoT devices and SC API is the container running on **Blockchain API Gateway** in FogNet. The containers were deployed in the EdgeNet, with resources being limited in 200 Mhz of CPU and 200 MB of RAM, running **IoT Edge API Gateway**, representing the IoT devices. In the AppNet, we deploy SC API. As a SC API, we use the IoT-Framework Engine [41] application from work [6] and its IoT-Framework-Gui [42] frontend.

**Figure 10:**
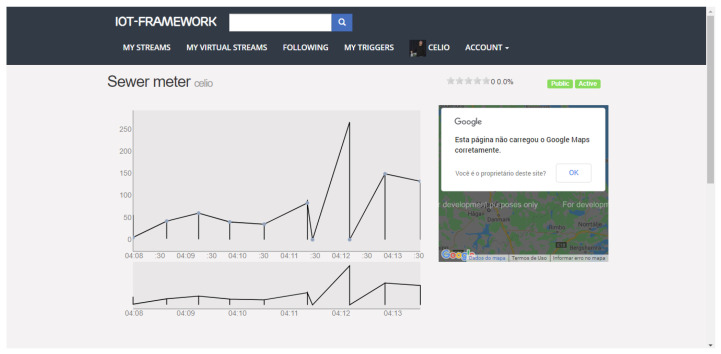
IoT-Framework-Gui.

We chose this project to represent an SC API due to its components being developed to scale. The core of its API is developed in Erlang, a platform that has shown promise in products that need to meet a large number of requirements. Figure 10 shows its web interface, with a graph resulting from data that were collected from our testbed’s IoT devices. In detail, a graph of density information in public sewage.

AppNet is an isolated network and, to contact it, EdgeNet always needs access to FogNet, which has contact with the internet, EdgeNet, and AppNet,

As Blockchain, we use one of Ethereum’s public test networks, Ropsten.

Ropsten’s Blockchain is publicly accessible via the internet and it has the same resources contained in Ethereum’s main network. These Ethereum test networks help us to debug and test Dapps and their Smart Contracts.

The Dapp accesses Ropsten using the infura.io project. Infura provides instant, scalable API to Ethereum networks. The Smart Contracts of IoT Device Management was deployed in Ropsten while using Truffle to call Infura API. Regarding Web interface and registry of IoT devices, a version of the Web frontend of Dapp IoT Device Management developed in React was deployed in Heroku. To registry and validate devices is called the Smart Contracts in Ropsten using an endpoint in Infura API. The codes of the testbed are in [43], and the frontend deployed of Dapp IoT Device Management used in the testbed is in [44].

**Figure 11:**
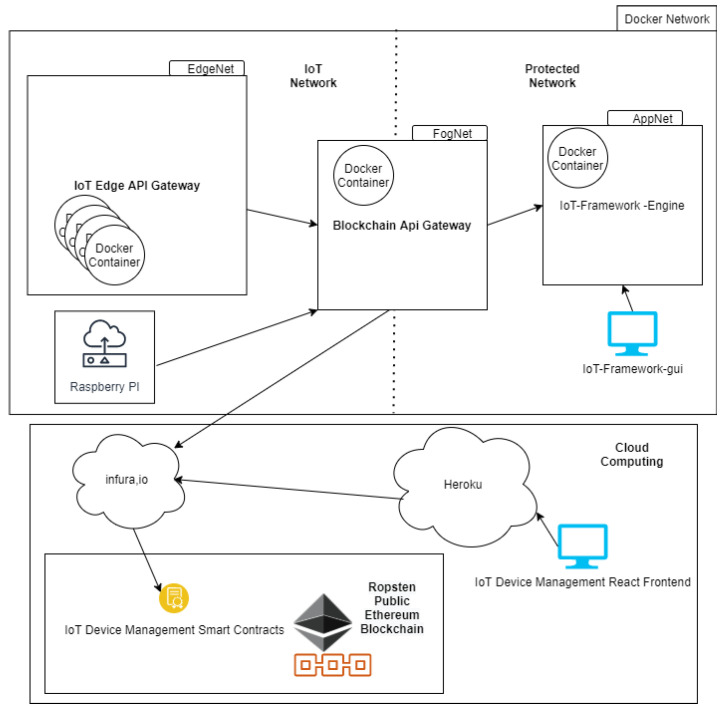
The Testbed network diagram.

Figure 11 details the network components and Docker containers used for the testbed.

We use real Raspberry Pi 3 IoT devices, Docker containers, and Desktops for the testbed, and install on these devices the **Iot Edge API Gateway**, On Raspberry, we use the Raspbian operating system. On Desktop and Containers, we use Ubuntu.

### 7.2. Results

We collected some experiments in scenarios using these devices for sending messages to understand the impact of times on the components of our ApI Gateway, on its layers, and the receipt of messages by the SC APP in scenarios of multiple devices generating payloads.

**Figure 12:**
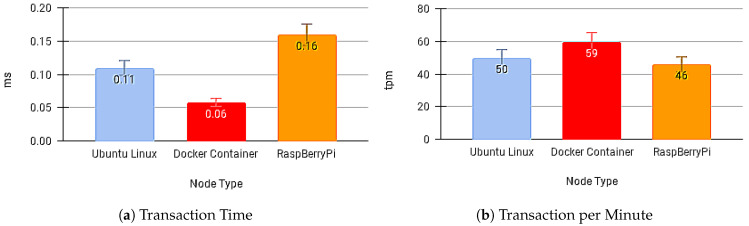
Results without Blockchain API Gateway.

Figure 12a,b present the transaction times for sending the message and the number of transactions per minute with no API Gateway.

**Figure 13:**
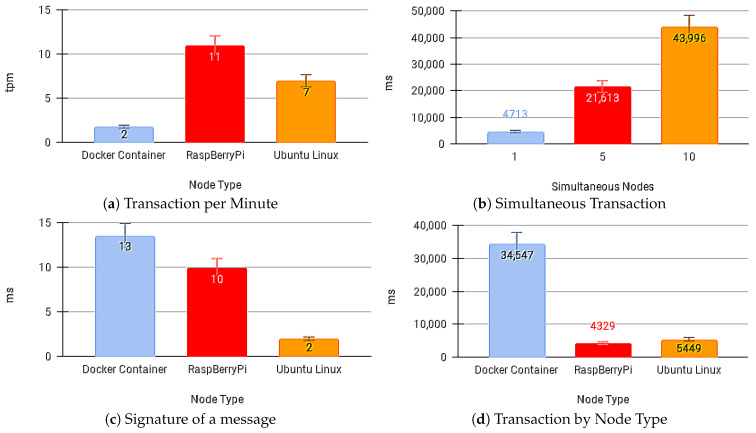
Average Time using the IoT Edge API Gateway.

Figure 13b shows the average time for a transaction on the IoT API Edge when considering the shipping competition with other devices on the network. Figure 13d shows the average transaction time on the IoT API Edge by device type. Figure 13c shows the average time to IoT API Edge sign messages by technology. Figure 13a shows the number of transactions per minute achieved on devices using IoT API Edge to send payloads to the IoT Blockchain API Gateway and receive a response from the SC App API.

**Figure 14:**
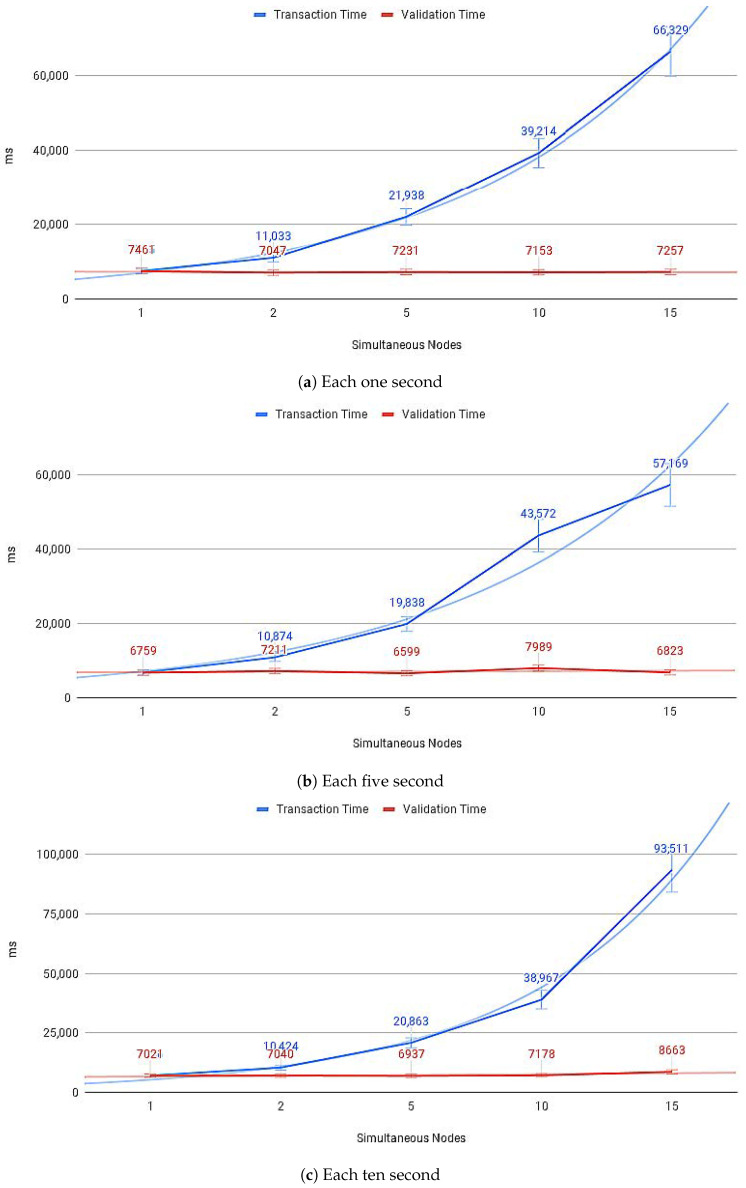
IoT nodes sending payloads.

**Figure 15:**
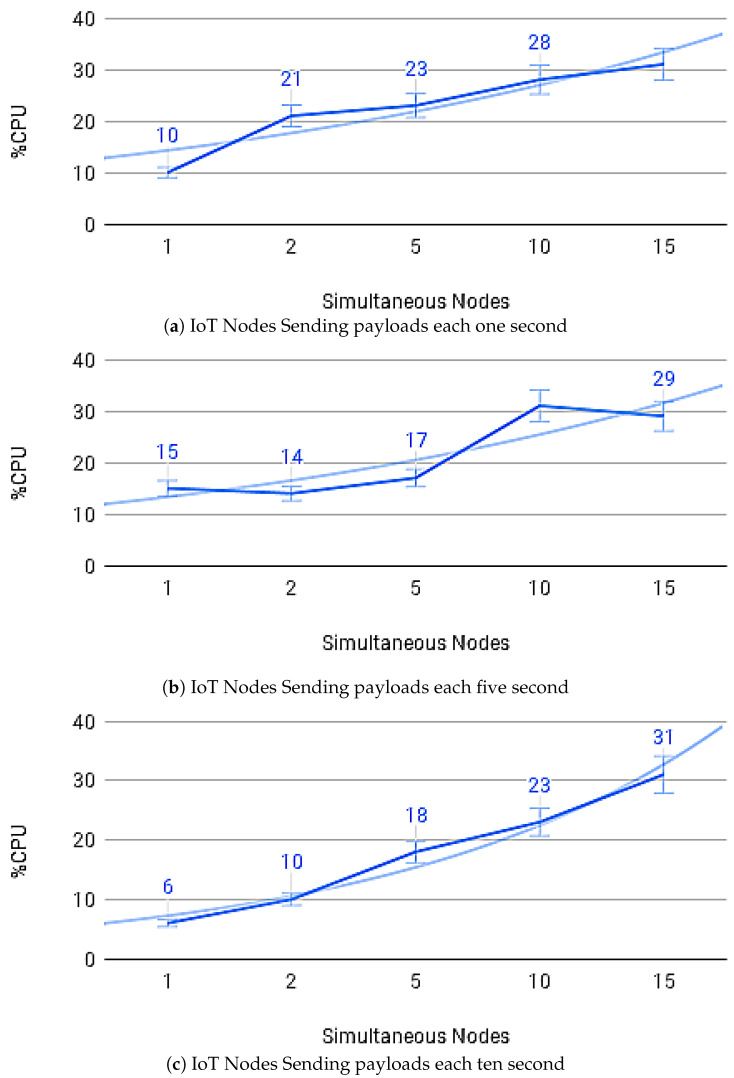
CPU usage.

**Figure 16:**
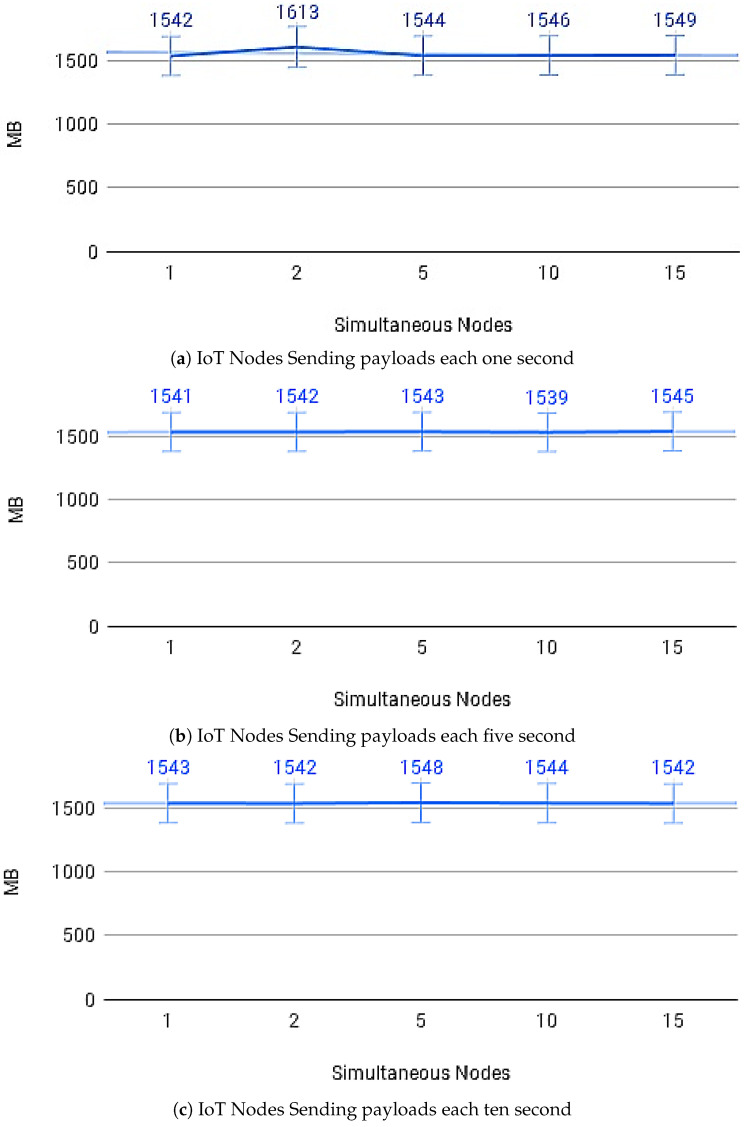
Memory usage.

Figure 14a–c show the average payload transfer and validation times on the IoT Blockchain API Gateway when considering the competition for sending with other devices on the network with one, five, and ten devices running the IoT Edge API Gateway. Figure 15a–c show the average of CPU use and Figure 16a–c memory use, during transactions in the IoT Blockchain API Gateway.

## 8. Discussion

Despite having SC Apps as a motivator, we can also apply our work to the vast majority of IoT use cases in Industry 4.0 that require a data origin guarantee.

We do not stress the possibilities of attacks in our scenario and thoroughly investigate the possible security holes in the implementation and architecture. Our API Gateways is an initial motivator for discussion to provide security and authenticity at the data source coming from the network’s edge.

The routines for consulting the Blockchain have no significant interference in the transactions that depend on it to verify metadata’s authenticity. We concluded that applications could use smart contracts that do not generate writing to the Blockchain without prejudice in performance.

The open-source Blockchain Ethereum is growing in popularity as both a cryptocurrency and a platform to develop Smart Contracts. It has a good part of the attributes needed to develop Dapps and projects like IPFS. These projects aimed at decentralized development promise to change the paradigm of the next generation of applications in a Web3. The tools that have been developed by the community for Ethereum are continually changing. Furthermore, its integration with projects is natural due to the immense adherence of open source developer communities to the project. During our research, it was possible to use Ethereum’s development networks, like Ropsten, enabling the experience of using a public Blockchain network for our implementation in the same molds of cryptocurrency applications.

Blockchain has not yet been extensively tested in non-crypto currency or financial scenarios. Use cases, like SC Apps, create a strong need for intense development of new and disruptive IoT applications. This phenomenon creates an urgent need to investigate and extend many of the points covered in superficiality by this research. These investigations’ importance becomes more relevant when there is a hypothesis that IoT applications will be globally implemented in the coming years, because the COVID19 crisis causes technological acceleration. SC tools that are implemented with security models discussed in our research may help in the next health crises, providing reliable information from institutions, validating data, and identifying their origins.

From our testbed’s transaction times, we can conclude that, although we cannot carry out transactions in real-time, the solution has strong adherence in applications where the times for sending payloads via IoT have a frequency of hours or days.

In some SC Apps, the government agencies may not donate the device, which the citizens have acquired. It improves the risks for the use of devices with technical characteristics unknown and out of a standard. A previous registration of this unknown device and the extraction of the hash of its firmware for validation can help to minimize these risks. It demonstrates the relevance of we deep in our research.

This security strategy can help to identify and validate the origin outside the IoT domain. For instance, the need to verify a news origin and identify the author is a common problem in our current society in the fight against fake news.

We will investigate strategies for load balancing and automatic scheduling in future work, which would validate our proposal in a production environment.

One of our ideas for future works will be to investigate the possibility of integrating our solution with the API Gateway of the cloud industry, like Apigee, providing modules and plugins utilizing Blockchain as background.

## Figures and Tables

**Table 1:** List of related works.

Techniques	Problems Addressed	Contributions
Blockchain [12]	Survey	Concentrate works in information systems
Blockchain and IoT [13]	Database for IoT Apps	A Simple Mechanism to Blockchain based Database
Blockchain, Smart Contract and IoT [14]	Automate complex processes	Identify solutions and workarounds in the combination of Smart Contract, Blockchain and IoT
Blockchain, SC and IoT [15]	Information security and privacy of IoT	a security framework that integrates the blockchain technology with smart devices
Blockchain, SC and IoT [16]	Blockchain-assisted information distribution system for the IoT	Design of the system
Blockchain and Fog Computing [17]	Secures sensitive data with encryption, authentication	Ensure improved security features through Blockchain technology
Blockchain and Fog/Edge Computing [18]	Applications of Blockchain-enabled fog	unveils the working relationship of Blockchain and the fog/edge
Blockchain, IoT and Edge Computing [19]	Cooperation and collaboration of recurses	An incentive-based mechanism to offer a reward for the participant in the process using Blockchain
IoT, Kubernetes and Fog Computing [20]	Analytics applications without sending everything to the data centers	iA analysis platform in the Fog Computing using Kubernetes
Blockchain and IoT [21]	Tracking and revocation of malicious users	Blockchain access control scheme with traceability and revocability in IIoT for smart factories
Fog Computing and IoT [22]	Availability of application-layer	MQTT-driven IoT-Fog integration
SC, IoT and Edge Computing [23]	Artificial Intelligence (AI) in Edge	PnP-AI and its impact in the SC
Blockchain, IoT and Edge Computing [24]	Integrates IoT with Edge Computing and Blockchain	Proposed a model designed for a scalable and controllable IoT system
Blockchain, Smart Contract and IoT [5]	IoT identity, security and interoperability	Systems users, entities, register devices using Smart Contract with and control information in a web interface

**Table 2:** List of Smart City (SC) Apps.

SC App	Problem	Solution
**City Trafficy**	Firmware update cycle depending on vehicle maintenance or depreciation date	Security layers that work independently of firmware security features
**Air Quality**	Physical access in high altitude places cause delays in updated firmware and battery changes	LoW-power devices and a security layers that work independently of firmware security features
**Temperature**	Physical access in high altitude places cause delays in updated firmware and battery changes	LoW-power devices and a security layers that work independently of firmware security features
**Analysis of Sewers**	Physical access in hard access places cause delays in updated firmware and battery changes	Security layers that work independently of firmware security features
**Rain Fall**	Physical access in high altitude places cause delays in updated firmware and battery changes	LoW-power devices and a security layers that work independently of firmware security features
**Tourism**	Dissemination of tourist information and the price of false public attractions affecting the city’s reputation, use of tourist data	Security layers that sign and verify the origin of messages
**Public Health**	False dissemination of citizen health data	Sign and verify the origin of messages
**Public Services**	False dissemination of citizen and service data	Sign and verify the origin of messages

**Table 3:** List of atributes and parameters used in Testbed.

API Gateway	Type	SO	Hardware	Parameters	Network
**Blockchain API Gateway**	Docker Container, Pc	Linux	Intel	simultaneous transmiting nodes, average Time To Transaction, transactions Per Minute. average time to validation, validations per minute, CPU Average, Mem Average, time To New payload	FogNet
**IoT Edge API Gateway**	Docker Container, Raspberry, Ubuntu Linux	Raspbian, Linux	Intel, ARM	average time to transaction, transactions per minute, average time to assign, signatures per minute	EdgeNet
**none**	Docker Container, Raspberry, Ubuntu Linux	Raspbian, Linux	Intel, ARM	average time to transaction, transactions per minute	EdgeNet

## Data Availability

Data available in a publicly accessible repository that does not issue DOIs Publicly available datasets were analyzed in this study. This data can be found here: https://docs.google.com/spreadsheets/d/101CTHrdj_N7Q4RT7_wUzrdR1RcLUEf0UMUoI2_8eCds/edit?usp=sharing.
